# Measuring the Safety of Excreta Disposal Behavior in India with the New Safe San Index: Reliability, Validity and Utility

**DOI:** 10.3390/ijerph110808319

**Published:** 2014-08-15

**Authors:** Marion W. Jenkins, Matthew C. Freeman, Parimita Routray

**Affiliations:** 1 Department of Civil and Environmental Engineering, University of California Davis, One Shields Ave., Davis, CA 95616, USA; 2 Environmental Health Group, Faculty of Infectious and Tropical Diseases, London School of Hygiene and Tropical Medicine, Keppel Street, London WC1E 7HT, UK; E-Mail: parimita.routray@lshtm.ac.uk; 3 Department of Environmental Health, Rollings School of Public Health, Emory University, Atlanta, GA 30322, USA; E-Mail: mcfreem@emory.edu

**Keywords:** latrine use behavior, open defecation behavior, child feces disposal, gender, measurement methods, attitudes, behavioral determinants, India

## Abstract

Methods to assess household excreta disposal practices are critical for informing public health outcomes of efforts to improve sanitation in developing countries. We present a new metric, the Safe San Index (SSI), to quantify the hygienic safety of a household’s defecation and human feces disposal practices in India, where behavioral outcomes from on-going public expenditures to construct household sanitation facilities and eliminate open defecation are poorly measured. We define hygienic safety of feces disposal as capture in a hygienic sanitation facility. The SSI consists of 15 self-report items and two sub-scales, Latrine Use Frequency and Seven-Day Open Defecation Rate. Households are scored on a standardized scale from 0 (no defecation safely captured) to 100 (all defecation safely captured). We present results of a pilot study in Odisha, India to apply the Index to assess excreta disposal behaviors among rural households and evaluate the reliability and validity of the Index for estimating the rate of correct and consistent sanitation facility usage of household with an improved latrine.

## 1. Introduction

Open defecation and unsafe excreta disposal continue to be widespread in rural India with major public health and economic consequences [1,2]. Since 2000, the Indian government has financed the construction of more than 95 million household latrines under the Total Sanitation Campaign (TSC) and provided thousands of community awards under Nirmal Gram Puraskar to stimulate behavior change [3]. A key goal has been to achieve 100% sanitized communities in which everyone uses a sanitary latrine and open defecation has ended [3]. Despite these substantial efforts, studies continue to indicate widespread sub-optimal use of latrines in rural India [4,5,6,7,8,9,10], undermining a key purpose of sanitation investments to contain fecal pathogens and improve public health.

The full extent, persistence, and characteristics of non-use of household toilets across rural India are poorly understood in part because of a lack of validated metrics and inconsistent unreliable methods to assess latrine usage and the absence of open defecation. With a renewed focus on ending open defecation by 2022 under the new Nirmal Bharat Abhiyan strategy for rural sanitation [3], there is an urgent need for reliable tools to quantify consistent and sustained sanitation use by all family members beyond simply calculating coverage rates as has been done to date. Towards this end, we set out to develop and test a novel metric, the Safe San Index, to quantify the safety of household excreta disposal practices in India, based on self-report responses.

Self-report of healthy/risky behaviors, though prone to social bias and recall error, is methodologically compatible with current household survey methods employed widely to track and assess sanitation access at household and population levels. Self-report as currently employed in the sector typically entails few questions, for example the Joint Monitoring Program for Water Supply and Sanitation asks what kind of facility members of the household usually use for defecation [11]. Another common question format in project-based surveys may ask how often members (in general) use the latrine. Such questions yield little information about the behaviors of individual members at specific times or the consistency of those behaviors. More robust self-report quantitative tools, specifically to measure consistent use of sanitation facilities by all members, verify open defecation free status, and measure progress (or lack thereof) towards achieving safe household excreta disposal in India as the critical health behavior outcome from sanitation investments are needed.

We considered but rejected two other potential measurement methods, namely structured observation and smart sensors. By avoiding recall, structured observation yields more accurate estimates of visually observable behaviors that occur in stable, predictable locations, but often capture only a very small sample of events [12]. Structured observation has largely been limited to measuring safe disposal of infant and young child feces by the caregiver [13,14] and is prone to other biases, including the Hawthorne effect [15,16]. Given varied places in and around a community for open defecation over the course of a day, from day to day, and as seasons change, and its private and hidden nature, direct observe of other members’ defecation places is impracticable and imprecise.

Electronic devices to detect and record motion and other physical phenomena hold promise for overcoming some of the challenges with structured observation and self-report of latrine use behavior [17]. Installed in an object (e.g., latrine, soap bar), sensors however are unable to collect data at the individual level or capture disposal that occurs elsewhere such as open defecation [15]. In theory, objects and individuals could both be wired to collect detailed individual-object interaction data. Besides high cost, a major challenge to widespread use of smart sensors is the significant data processing, calibration, and interpretation work needed to make sense of signal patterns to identify behaviors of interest. On a practical level, structured observation and smart sensors entail much greater time, effort, expense, and field staff skills and expertise, per sampled household, severely limiting their broad application.

The goal of this research was thus to develop and test a novel instrument to assess safe excreta disposal behavior in the home for application in rural India to monitor and evaluate sanitation projects and programs aimed at ending open defecation, with the following characteristics:

- Provide a fine-grained interpretable quantitative measure of the degree of safety of defecation and fecal disposal behaviors of individuals in a household using low-cost self-report survey methods;
- Allow for reliable, accurate and comparable quantitative estimates over time and space of the extent of use of household sanitation facilities for all human fecal disposal events;
- Offer a reliable household survey-based method to quantify open defecation activity, track changes in activity, and help verify its end in target communities.

In this paper, we introduce the Safe San Index (SSI) and present the results of a pilot study in Odisha State to test and evaluate the Index as an instrument to quantify safety of household excreta disposal practices in India.

## 2. Methods

We describe the two sub-component scores—Latrine Use Frequency and Seven Day Open Defecation Rate—and 15 self-report items comprising the SSI and the research approach to assess its reliability and validity. We then present the design of the pilot study to test and evaluate the SSI.

To our knowledge, the SSI is the first attempt to develop a metric to quantify the hygienic safety of a household’s defecation and human feces disposal behaviors, accounting for intra-household heterogeneity in members’ practices at different defecation and disposal occasions. Safety here is defined as disposal of feces in a household sanitation system, one that presumably meets established hygienic sanitation norms. Determination of system hygienic safety would be a separate evaluation task requiring different metrics and methods [18,19] and is outside the scope of the SSI’s focus on household sanitation behavior and optimal use of existing infrastructure. Given a representative household sample, the SSI has been designed to provide a quantitative estimate of the proportion of human fecal waste generated in a community which is safely disposed, varying from 0 (no defecation safely captured) to 100 (all defecation safely captured).

### 2.1. Preliminary Exploratory Insights

Eight exploratory focus group discussions (FGDs) were conducted with community members with a household latrine and one with front line NGO workers involved in implementing the TSC in coastal Odisha. TSC evaluations and sanitation studies in India were reviewed to explore dimensions of household latrine use, continued open defecation behavior, and associated attitudes. Several key observations emerged to guide SSI development for this context:

- Defecation places and practices can differ substantially between individuals in the same household (*i.e*., men *vs*. women, married *vs*. unmarried, younger *vs*. older adults, young children *vs*. adults), change with time of day, and vary within and across seasons in relation to changes in the nearby landscape, agrarian cycles, and physical and social opportunities and constraints on the choice of available places in the community.
- Safe disposal of feces in the latrine or toilet of members incapable of using the toilet, whether due to age, illness, or infirmity, is an important and understudied dimension of household sanitation, but is critical for achieving sanitary conditions given that diarrhea and its enteric pathogens are likely to be produced by the young and ill. FGDs indicated that even in households with long established and consistent latrine use, adults were unaware of the importance of safely disposing of these persons’ feces in a latrine and found the idea inconvenient.
- Very little stigma is attached to open air defecation, and the practice is associated with certain advantages and benefits over using latrines. It thus persists alongside and despite ownership and regular use of a latrine.

A metric would thus need to include feces disposal practices for those physically unable to use a sanitation facility, disaggregate and assess defecation places at the individual level, and consider different times for defecation in the day and night. It would also need to focus on a narrow temporal window to reduce recall bias. Moreover, questions would need to directly ask about both latrine use and open defecation activity, as the two behaviors are regularly practiced by a large portion of individuals with access to a latrine in both rural [6,8,9,10] and urban areas [20,21] of India.

### 2.2. Safe San Index Components and Measurement

The first component—Latrine Use Frequency (LUF)—measures the average frequency of latrine use among individuals in the household across multiple defecation and feces disposal opportunities. Scored from 0 to 100, LUF captures individual behavior grouped by demographic and accessibility categories emerging in the FGDs. It comprises 11 items measured by 12 self-report questions (see Table 1). The second component—Seven-day Open Defecation Rate (ODR7)—captures the amount of recent open defecation activity by the household’s population. Also scored 0 to 100, ODR7 estimates the daily prevalence of open air defecation on each of the past 7 days by individuals in the household. It is constructed from 4 items measured by 7 self-report questions (see Table 1). Both components of SSI are necessary to measure whether safe defecation and feces disposal occurs all of the time and each has value on its own. LUF quantifies the propensity (or likelihood) of members of the household to use the sanitation system at each defecation and feces disposal opportunity, while ODR7 is the actual estimated daily rate of unsafe open air defecation by members during the last seven days.

**Table 1:** Safe San Index items, scoring, and computation.

Q #	Item #	Survey Question	Response Options (Score Value)
1	-na-	How often do you (respondent) personally use the toilet to defecate?	Never (1) Sometimes/Occasionally (2) Usually/Mostly (3) Always (4)
2	LUF 1	How often do the elders (over 60 years of age) in your household use the toilet to defecate?	No members/ others besides respondent (0) Never (1) Sometimes/Occasionally (2) Usually/Mostly (3) Always (4)
3	LUF 2	How often do the (other) married women who are not elders, use the toilet to defecate?	(same)
4	LUF 3	How often do the (other) unmarried women (over 15 years old) who are not elders, use the toilet to defecate?	(same)
5	LUF 4	How often do the married men who are not elder use the toilet to defecate?	(same)
6	LUF 5	How often do the unmarried men (over 15 years old) who are not elder use the toilet to defecate?	(same)
7	LUF 6	When school age children in your household are at home, how often do they use the toilet to defecate?	(same)
8	LUF 7	For young children who are too young to be able to use the toilet, after they defecate on the ground in your courtyard or in your house, how often do you put their feces in the toilet?	(same)
9	LUF 8	If a member of your household becomes critically ill * and must defecate in the dwelling or courtyard, do you throw their feces in the toilet?	Never (1) Sometimes/Occasionally (2) Usually/Mostly (3) Always (4)
10	LUF 9	When adult females (such as yourself) in your household have a need to defecate during mid-day, would they use the latrine?	(same)
11	LUF 10	When adult males in your household have a need to defecate during mid-day, would they use the latrine?	(same)
12	LUF 11	When any member of your household, whether adult or child, needs to defecate in the night, how often do they use the latrine?	(same)
13	-na-	On how many of the mornings of the last 7 days did you defecate in the open (e.g., field, bush, roadside, side of canal, back of house, *etc*.)?	No days (1) Some days (2) Most days (3) Every day (4)
14	-na-	On how many of the evenings of the last 7 days did you defecate in the open?	(same)
15	-na-	On how many of the last 7 days did you defecate in the open at noon time, or at night?	(same)
16	ODR7 1	Considering the routines of the other adults (over 15) in your household over the past week, on how many of the last 7 days did ANY other women in your household defecate in the open?	No (other) adult women (0) No days (1) Some days (2) Most days (3) Every day (4)
17	ODR7 2	On how many of the last 7 days did ANY men in your household defecate in the open?	No (other) adult men (0) No days (1) Some days (2) Most days (3) Every day (4)
18	ODR7 3	Considering the routines of children (15 and under) in your household during the past week, on how many of the last 7 days did school age children in your household defecate in the open?	No school age children (0) No days (1) Some days (2) Most days (3) Every day (4)
19	ODR7 4	On how many of the last 7 days did any pre-school age child in your household defecate in the open?	No pre-school children (0) No days (1) Some days (2) Most days (3) Every day (4)
**Computation Steps**			
LUF Index component		If respondent’s response (Q1) is lower than the LUF response for their specific demographic (*i.e.*, LUF item 1, 2, 3, 4 or 5), replace the demographic response value with the Q1 response value. Sum LUF items 1to 11 to compute a raw LUF total score (possible raw range 5–44). Count #of LUF items with non-zero score value (possible range 5–11). Standardize the raw LUF total score using the ratio of non-zero to total (11) items (possible standardized range 11–44). Convert the standardized LUF total to an equivalent 0–100 scale LUF Index score.	
ODR7 Index component		Determine respondent’s overall score as maximum of Q13, Q14, and Q15. If respondent’s overall score is higher than the ODR7 response for their demographic (*i.e.*, ODR7 item 1 or 2), replace the demographic response score with the respondent’s score. Sum ODR7 items 1to 4 to compute a raw ODR7 total score (possible raw range 1–16). Count #of ODR7 items with non-zero score (possible range 1–4). Standardize the raw ODR7 total score using the ratio of non-zero to total (4) items (possible standardized range 4–16). Convert the standardized ODR7 total to an equivalent 0–100 scale ODR7 Index score.	
Safe San Index		Calculate the ‘No-ODR7’ Index score as 100 minus the ODR7 Index score. Multiply the LUF Index score by the No-ODR7 Index score and divide by 100 to compute the Safe San Index score.	

Note: * The notion is “so ill”, that is bed-ridden or in a state of weakness that the person is unable to walk to the latrine or to an open defecation area away from the house.

SSI responses are best obtained from the individual most likely to know the current defecation and feces disposal places of both children and other members living at home, typically the female head of the household in rural India. In her absence, the male head or another senior member most likely to know can be interviewed. LUF items are self-assessed on a 4-point scale from “never/rarely” (value = 1), “sometimes” (value = 2), “usually/mostly” (value = 3), or “always” (value = 4). Non-applicable items receive a zero value; for example, when the household has no elderly members, item LUF 1 (Table 1) is scored zero. To minimize potential social bias in computing scores, the less safe of the respondent’s own reported behavior and that reported of her (his) demographic category is used (step #1, Table 1). Successive steps in constructing a household’s LUF score consist of summing item values, adjusting the raw total score to produce a standardized score using the ratio of relevant (non-zero) to total items (11), then scaling the standardized score to its equivalent 0–100 scale index value (details in Table 1). For example, if a household responded “sometimes” (value 2) to all applicable LUF items, their resultant LUF score would be 33. Thus, household scores correspond to LUF levels “never/rarely” (LUF < 33), “sometimes” (33–66), “usually/mostly” (67–99) and “always” (100) use.

Similar procedures are applied to measure and construct the ODR7 score (see Table 1). Items are self-assessed on a 4-point scale of “no days” (value = 1), “some days” (value = 2), “most days” (value = 3), or “everyday” (value = 4), with non-applicable items receiving a zero value. An ODR7 score of zero means no members on any of the last 7 days defecated in the open, while a score of 100 indicates every member defecated in the open at least once on each of the last 7 days.

### 2.3. Evaluating Index Reliability

The SSI uses self-report, and thus depends on obtaining reliable (repeatable) and truthful responses from a respondent. To test SSI reliability, household scores were measured at two visits, separated by approximately four weeks. Four weeks was chosen to minimize respondent recall of earlier responses and minimize genuine temporal changes in defecation behavior. To control for true changes in sanitation behavior between the two visits, respondents were asked to self-assess their household’s current defecation places in terms of similarity/difference compared to places used for defecation one month prior and to places used in other seasons. Index scores are sensitive to the number and demographic categories of members, so information was collected on household size and membership at each visit.

Index score reliability was assessed by comparing the household’s SSI score and its sub-components at the two visits using the non-parametric Spearman’s rank correlation test (ρ) and the paired samples Wilcoxon signed rank test of agreement. In test-retest reliability comparison, measurement conditions should be stable, thus agreement was examined separately for households with stable (*i.e.*, unchanged membership who characterized their current defecation places relative to a month ago as “mostly the same” *vs.* “somewhat the same”, “somewhat different” or “mostly different”) and unstable test-retest conditions. Agreement of the household’s LUF level was also assessed, applying ordinal by ordinal tests (Sommer’s d, Kendall’s tau b, Gamma) and Cohen’s Kappa for nominal agreement. Values of 1 indicate perfect agreement for Spearman’s ρ and ordinal by ordinal tests, while for the Wilcoxon test it is 0. Near perfect agreement is indicated by a Kappa > 0.80, while a Kappa between 0.60 and 0.80 is considered substantial agreement [22].

To test truthfulness, respondents’ propensity to give socially desirable responses was measured using the Social Desirability scale (SDS) [23]. SDS is an established psychometric scale of the tendency to respond in socially desirable ways, a limitation of self-report when measuring socially stigmatized or conditioned behaviors that may lead to over-reporting of behaviors perceived to be socially better or preferred and under-reporting of the opposite. Using the short 8-item scale [24], respondents’ SDS score was measured at enrollment. Higher SDS indicates greater propensity to give socially desirable answers. Correlation between a respondent’s SDS score and their LUF, ODR7, and SSI scores was tested using Spearman’s ρ to assess whether respondents with a greater tendency to give socially desirable responses (higher SDS) were more likely to report “better” behavior (*i.e.*, more latrine use, less open defecation).

### 2.4. Evaluating Index Validity

**Figure 1:**
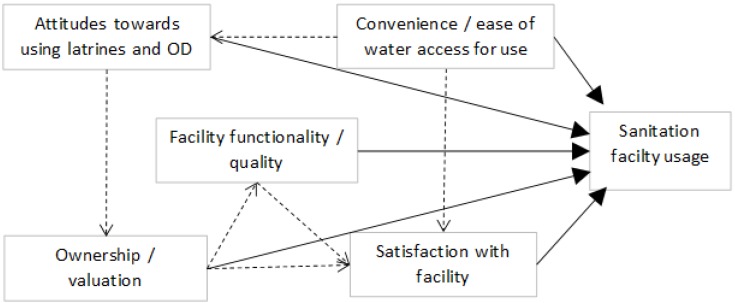
Factors that influence latrine use rates and open defecation behavior identified from India and global literature on latrine adoption and use.

To examine validity of the SSI as an accurate quantitative measure of the rate of latrine use for defecation and disposal events, we identified five conditions that increase latrine use and reduce open defecation behavior (Figure 1) based on factors reported in literature from the Indian context [4,5,6,7,10,20,21,25] and elsewhere [26,27,28,29,30,31,32,33,34] and confirmed qualitatively in the FGDs. 

The five conditions comprise:

1. Convenience/ease of water access for using the latrine. Water is necessary for operation, use and cleaning of water-based pour flush latrines and where anal cleansing with water is practiced, the case in most of India. Water access for bathing at the latrine may be additionally important where cultural practices of body bathing and washing one’s attire after defecation or contact with human feces and before engaging in “clean” activities are ingrained, as observed in the study area.
2. Positive liking or preference for using a latrine over open defecation reflected in more positive *versus* negative beliefs, attitudes and social norms towards latrine usage.
3. Ownership and valuation of the latrine facility is evidence of motivation to want and use the facility as well as invest in its continued operation and maintenance.
4. Functionality and quality of the latrine facility must be adequate, acceptable, and appealing to users for correct and consistent usage to occur.
5. Satisfaction with the latrine facility provides a robust overall indicator of how well the facility meets the household’s needs and expectations.

Survey questions and observations of the latrine facility were used to measure multiple indicators for each condition. Positive attitude towards using latrines over open defecation was quantified using responses to semantic differential (SD) and Likert scale (LS) items on a simplified 3-point scale. Item content was generated directly from FGD content and pretested, and an attitude score constructed by summing item responses. The SD and LS attitude items and scoring are detailed in supplemental material.

Associations between a household’s LUF, ODR7 and SSI scores and predictive conditions were tested for expected direction, or trend in the case of ordered level variables, and strength (significance). Bivariate analyses applied Spearman’s ρ for continuous variables (e.g., positive attitude SD score), one-way ANOVA of group mean differences for binary nominal variables (e.g. fully constructed: yes, no) and the linear trend test of ANOVA group means for ordered variables (e.g., satisfaction level).

### 2.5. Pilot Study Setting, Participants and Sampling Approach

Participants were from randomly selected households living in eight rural villages in Puri District, Odisha State, India. Villages located within a three hour drive of Bhubaneswar City were purposively selected to capture four dominant household sanitation infrastructure situations (strata) common in rural India: no latrines; latrines subsidized by the Government of India (GOI) via the TSC without further improvements made by the owner (“GOI latrine”); GOI latrines with self-financed improvements made by the owner (“GOI+ latrine”), and latrines completely self-financed by the owner without any NGO or government subsidy or intervention (referred to “SF latrines”). All latrines in study villages were pour flush to a pit or to a septic tank. Only operable latrines were considered. The three latrine investment strata efficiently captured variations in facility design, quality, functionality and sense of ownership. Households without any latrine served as the reference to which latrine owner ODR7, LUF7 and SSI scores could be compared and to test data collection and measurement at one extreme of the index sub-component scales.

A sampling frame of all households with a child under age 5 for each village was created, including latrine ownership (none, GOI, GOI+, SF). From 20 to 26 eligible latrine households in each investment strata were randomly selected across villages, with the balance selected from non-owners, for a total of 120 enrolled households.

### 2.6. Data Collection

Three rounds of data collection occurred from early September (end of monsoon season) through end of December (beginning of winter season), 2011. At baseline, we obtained informed written consent to participate in the study and administered the baseline survey (BL). During the first follow-up visit (F1) four weeks after BL, we administered the SSI survey questions (Table 1). A second follow-up visit (F2) four weeks later repeated the SSI survey. Surveys were verbally administered in Oriya to the same female head of household at each round whenever possible. Field work was conducted by a small team of 4 trained and experienced enumerators supervised in the field by a member of the research team, all of whom were native Oriya speakers, following translation and pre-testing of the questionnaire.

The BL included socio-economic information, water access, the eight-item SDS, the attitude statements, and data on predictive condition variables. The F2 survey included questions about the stability (similarity) of defecation places “now” with those used in the prior month and household size and membership were recorded at each visit. To gain insight on seasonal variations, we asked how often members defecate in grain fields after harvest (starting late December, early January). The period following harvest is when FGD participants said latrines were least used because weather is good, people have more time, and nearby agricultural land is free of standing monsoon water and rice cultivation, providing plentiful convenient places for open air defecation. The ethics committees of the London School of Hygiene and Tropical Medicine and Xavier Institute of Management Bhubaneswar approved the study.

## 3. Results and Discussion

The same person was interviewed at 95 households out of the 112 who completed all three rounds of the study. A small number of households dropped out (n = 5) or were unreachable at F1 (n = 1) or at F2 (n = 2).

### 3.1. Sample Description

Table 2 shows characteristics of study households. Mean size of study households was seven, including one child under age five. A majority of study households obtained their main income from farming (53%), were headed by a male (89%), were Hindu (100%), had a below-poverty-line card (58%), and belonged to a scheduled or other backward caste (60%). In 47% of households, the family’s female head was illiterate compared to only 10% illiteracy among male heads. These statistics largely reflect the rural population in Puri District where the pilot study was conducted.

**Table 2:** Pilot study household descriptive statistics.

Characteristic	N	Mean [SD]	Min–Max	n (%)	n (%)
Household size (F1)	114	6.9 [3.1]	2–19		
Household size (F2)	112	6.9 [2.9]	2–18		
Children < 5 (F1)	114	1.0 [0.84]	0–6		
Children < 5 (F2)	112	0.97 [0.73]	0–4		
Female respondent (F1)	114			112 (98)	
Gender of household head	114				
*Male*				101 (89)	
*Female*				13 (11)	
Hindu religion	114			114 (100)	
Main income source	114				
*Farming*				60 (53)	
*Service holder*				15 (13)	
*Business enterprise*				12 (10)	
*Day or shared labor*				17 (15)	
*Other*				10 (9)	
Education of male & female head				Male n = 111	Female n = 114
*Illiterate*				11 (10)	49 (43)
*Literate w/o formal schooling*				10 (9)	9 (8)
*Some primary (1st-4th)*				26 (23)	28 (25)
*Primary completed (5th)*				31 (28)	18 (16)
*Secondary completed (10th) or above*				33 (30)	10 (9)
Below poverty line (BPL) card (verified)	114			66 (58)	
Caste—household head	114				
*Scheduled caste*				15 (13)	
*Other backward caste*				43 (47)	
*General*				53 (38)	
*Don’t know*				3 (2.6)	
Own cell phone	114			92 (81)	
Own agricultural land	114			83 (73)	
Own poultry/livestock	114			61 (54)	
Drinking water source	114				
*Piped water*				6 (5.3)	
*Hand pump (tube well)*				102 (90)	
*Protected dug well*				4 (3.5)	
*Unprotected dug well*				2 (1.8)	
Drinking water source location					
*In own dwelling*				12 (11)	
*In own compound*				21 (18)	
*Outside compound*				81 (71)	
Round trip time to off-site source (min)	81	14.8 [12.2]	1–60		
Bathing source = surface water (pond, river)	114			77 (68)	
1-way time to surface bathing site (min)	72	12.7 [10.3]	1–60		
Functioning latrine (F1) (all pour flush)	114			71 (62)	
Age of latrine (years)	71	7.1 [6.5]	2–30		

### 3.2. Water and Sanitation Conditions

Nearly all study households used an improved water source for drinking (98%), which was typically a public tube well. Despite near universal access to an improved source, 67% bathed at an off-site surface water location (*i.e.*, pond, river, canal) (Table 2). 

Of those who completed the study and F1 (n = 114), 42 had no latrine. Sampling was designed to obtain balanced random subsamples of owners across the three investment strata. However, baseline information showed that latrine owning households (n = 72) consisted of 5 GOI, 44 GOI+, and 23 SF latrines, with all but one still functioning at F1. Mean duration of ownership and operation of functioning latrines was 7 years, ranging from 2 to 30 years.

### 3.3. LUF, ODR7, and Safe San Index Household Scores

Among households without a latrine (n = 42), LUF was 0, resulting in SSI = 0, irrespective of ODR7. These households, as expected, had high ODR7 (mean [SD] = 94 [11.5]; median = 100). Ten had an ODR7 < 100 which can occur for a household without access to any sanitation facility if some members defecate less than once per day during the surveillance period, for example, when a member habitually defecates less than daily or an adult member is away from home for part of the week; if a school-age child defecates only at school on one or more of the days; or if the household has pre-school age children who defecate in a cloth, potty, or accidentally. Safe disposal of infant and young child feces accidentally deposited or collected in a potty, a caretaker behavior, is captured in LUF item 7.

**Figure 2:**
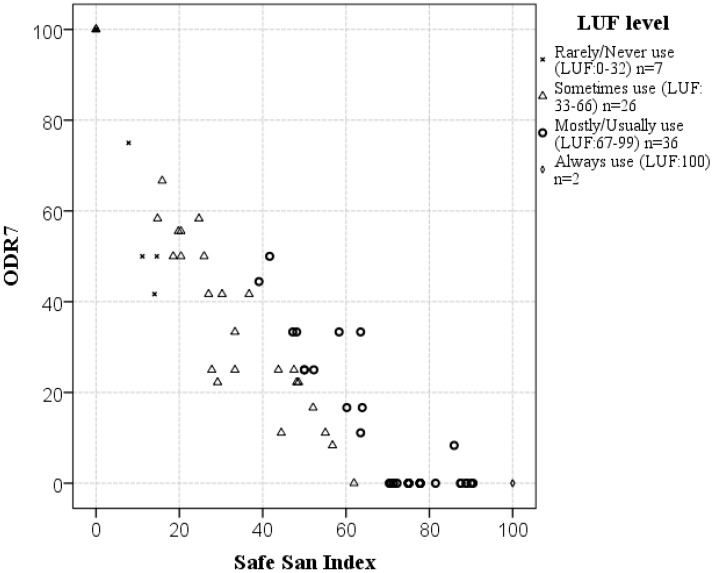
Extent of open defecation over the last 7 days (ODR7 score) of members of study households with a functioning latrine (n = 71), classified by LUF score, and plotted against their Safe San Index score (F1 data).

Among study households with a functioning latrine (n = 71 at F1), mean ODR7 was 27 (on average 27% of members openly defecated at least once a day), mean LUF was 64 (“sometimes” use range), and mean SSI was 51 (51% of defecation events safely captured). Only 2 households (3%) had ODR7 = 0 and LUF = 100, necessary for an SSI of 100. In some latrine owner households, every member defecated openly at least once on each of the last 7 days (ODR7 = 100), resulting in SSI = 0. The majority of owners (51% [n = 36/71]) had LUF scores in the “mostly” use range (67–99) where ODR7 ranged from 0 (no open defecation) to 50 (50% of members open defecated daily) and SSI ranged from 39 to 90. ODR7 variability within each LUF range is visible in Figure 2. Households who on average “sometimes” used their latrine (LUF: 33–66 range [n = 26/71]) showed the greatest variability in recent open defecation rates, spanning the full ODR7 range from 0 to 100. Scores at the second follow-up visit (F2) demonstrated the same relationships and patterns (not shown).

**Figure 3:**
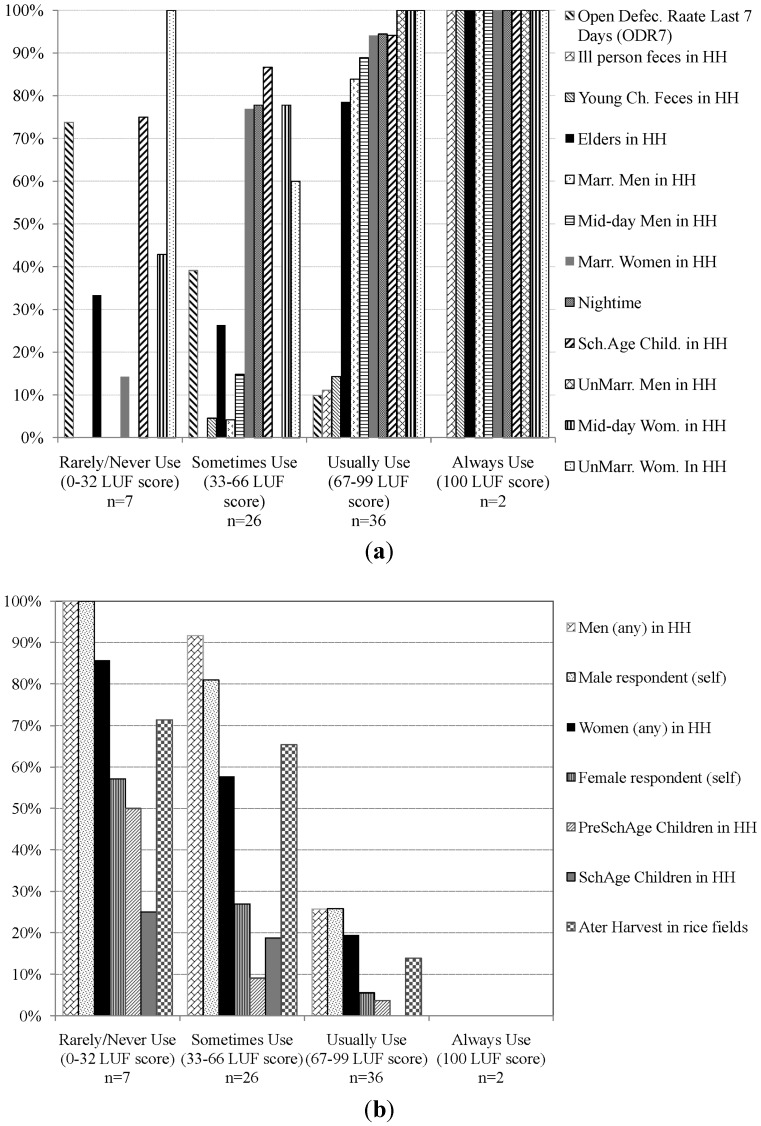
Intra-household and temporal variations in defecation behavior of study households with a functional latrine (n = 71), grouped by their LUF level (F1 data). (**a**) Frequency of “always use latrine” for each LUF item and average ODR7 score; (**b**) Portion reporting open defecation on “some”, “most” or “every” day of the last 7 days for each ODR7 item.

The demographic groups and occasions for which unsafe defecation and excreta disposal was most likely to occur in the study population of latrine owners are shown in Figure 3. Households with high LUF (*i.e.*, mostly/usually use [67–99 range] [n = 36]), reported high rates of consistent use among adults in all demographic categories and occasions (>80%), elders (>70%), and school age children (>90%). These high rates contrast with their very low rates of consistent use for safe disposal of young children’s (<15%) and ill persons’ feces (10%) (Figure 3a). These members’ feces were reported disposed on a manure, compost or garbage pile, or thrown into a pond or drainage instead of safely in the latrine. As household LUF dropped into the “sometimes” use range (33–66 [n = 26]), adult rates of consistent use declined sharply, remaining above 50% only for married and unmarried women, school age children, and defecation at night, with consistent use among unmarried women notably lower than among married women and school age children in these households (Figure 3a). In the “sometimes” use range, the proportion of men with consistent use, whether married or not, was near zero, and was also low for elders (<30%). Ill persons’ feces were never consistently disposed in the latrine and consistent safe disposal of young children’s feces fell below 5%. Among “rarely” use households (n = 7), exceptions of consistent use involved a rare individual unmarried woman, school age child, elder, or unmarried man. Consistent night-time use and safe feces disposal were nil. Average ODR7 rose from a low of 10% among members of LUF “mostly” use households to >70% among members of LUF “rarely” use households.

Consistent with intra-household patterns of latrine use behavior (Figure 3a), rates of open defecation during the last 7 days (Figure 3b) among school children were much lower than among adults, and men’s rates were always higher than women’s at each average LUF level below 100. ODR7 among pre-school age children able to defecate on their own was also low and matched the rate among school age children in households in the “sometimes” and “mostly” use LUF levels (<10% daily prevalence), but rose above 50% in households whose members on average “rarely” used their latrine.

Figure 3b also shows rates of any open defecation after harvest. Prevalence after harvest is similar to average ODR7 among both “rarely” (both 70%) and “mostly” (both 10%) use LUF households, but is higher than average ODR7 among “sometimes” use LUF households (>60% *vs.* <40%) suggesting latrine use patterns may be relatively stable in “rarely” and “mostly” use LUF households, but temporally variable and seasonally driven in “sometimes” use households.

### 3.4. Safe San Index Reliability

#### 3.4.1. Response Bias

We found no correlation between a respondent’s Social Desirability scale score (mean [sd] = 21.5 [2.5]) and their LUF, ODR7 or resultant SSI score (ρ = −0.006, −0.024, and −0.022, respectively) demonstrating a complete lack of statistical association (*p* ≥ 0.84) between the tendency to respond in socially desirable ways and reporting higher latrine use tendency or lower open defecation activity. 

#### 3.4.2. Repeatability

Scores at F1 and F2 are shown in Table 3. Mean ODR7 of the whole sample (n = 114) demonstrated high correlation (ρ = 0.84) and reliability (non-significant paired samples Wilcoxon test *p* = 0.16). To more fully evaluate reliability of ODR7, LUF and SSI, non-owners were removed from the analysis (LUF and SSI = 0 at both visits). Reliability was assessed for latrine owners with a functioning latrine at both visits, separately for those with stable (n = 38) and unstable (n = 31) measurement conditions. Results are reported in Table 3.

Among owners with stable household test-retest conditions, LUF scores demonstrated very high correlation (ρ = 0.90) and no statistical difference between F1 and F2 (Wilcoxon test *p* = 0.33). Reliability of ODR7 and SSI was also good, demonstrated by high correlations (ρ = 0.65 and 0.84, respectively) and no statistical difference in scores (Wilcoxon test *p* = 0.71 and 0.12, respectively). Ordinal tests of agreement between a household’s LUF level at F1 and F2 were all highly significant, and Kappa (0.72) indicated substantial 1:1 match (Table 4).

**Table 3:** Correlation and agreement of scores at the first (F1) and second (F2) follow-up visit.

Index	Mean Values Statistical Tests	All Households		Households with Functioning Toilet and Stable Test-Retest Conditions	Household with Functioning Toilet and Unstable Test-Retest Conditions
		N = 114 *		n = 38 **	n = 31 ***
ODR7					
	Mean [sd] F1	51.6 [40.3]		26.2 [33.5]	27.0 [24.1]
	Mean [sd] F2	56.8 [35.3]		27.2 [21.8]	39.7 [25.4]
	Spearman’s rho (p)	0.84		0.65	0.60
	Wilcoxon Signed Rank test **^+^** (p)	1.41 NS (0.16)		−0.37 NS ^§^ (0.71)	−1.84 NS (0.066)
LUF					
	Mean [sd] F1	38.7 [34.6]		65.5 [21.7]	61.6 [19.4]
	Mean [sd] F2	36.1 [34.5]		64.1 [22.4]	52.8 [26.1]
	Spearman’s rho (p)	-na-		0.90	0.79
	Wilcoxon Signed Rank test **^+^** (p)	-na-		0.97 NS (0.33)	2.61 (0.009)
SSI					
	Mean [sd] F1	29.9 [32.9]		53.7 [29.7]	48.1 [26.7]
	Mean [sd] F2	26.4 [30.4]		49.5 [24.8]	36.6 [29.8]
	Spearman’s rho (p)	-na-		0.84	0.75
	Wilcoxon Signed Rank test **^+^** (p)		-na-	1.56 NS (0.12)	−2.50 (0.012)

Notes: * 108 with valid ODR7 and SSI scores for both F1 and F2; ** 37 valid for ODR7 and SSI scores for both F1 and F2; *** 28 valid for ODR7 and SSI scores for both F1 and F2; **^+^** related samples test of agreement; accept null hypothesis that distributions (and mean) are the same when *p* ≥ 0.05; **^§^** non-significant result.

**Table 4:** Agreement of LUF level at F1 and F2 (Levels: rarely/never 0–32, sometimes 33–66, mostly/usually 67–99, always 100).

Measure	Statistical Test	Household with Functioning Toilet and Stable Test-Retest Conditions (n = 38)	
Ordinal × Ordinal		Statistic	*p*-value
	Sommers’d Symmetric	0.825	<0.00001
	Kendall’s tau b	0.828	<0.00001
	Gamma	1.00	<0.00001
Agreement			
	Kappa	0.723	<0.00001

Among the counterfactual group of owners with unstable household test-retest conditions, at F2 LUF was 9 points lower, ODR7 was 13 points higher, and SSI was 13.5 points lower than at F1, with paired samples statistically different for LUF and SSI (Wilcoxon test *p* = 0.009 and 0.012, respectively), and near significantly different for ODR7 (*p* = 0.066). F1 and F2 Spearman correlations were also notably lower in this subgroup with unstable measurement conditions (see Table 3). If household membership changed at F2, and added/removed members had different defecation behavior from others in the household, or if individuals truly changed how often they used their latrine from F1 to F2 due to temporal or other influences on choice of place and behavior, we would expect ODR7 as well as LUF and SSI scores to diverge, as the results demonstrate they do among this group.

### 3.5. Safe San Index Validity

Associations between LUF, ODR7 and SSI and the 19 variables measuring conditions predicted to increase latrine uptake and use (Figure 1) are shown in Table 5. Each score-variable association tested demonstrated a relationship in the expected direction (*i.e.*, positive for LUF and SSI, negative for ODR7). Associations were statistically significant (2-sided *p* < 0.05) for LUF and SSI with 17 of the 19 variables tested and all five conditions. ODR7 was significantly correlated with 12 of the 19 variables and all five conditions. Many associations and ordered trends were highly significant (*p* ≤ 0.001). These results provide good evidence of the validity of SSI and its sub-components as sound quantitative scale measures of latrine use, open defecation activity, and safe capture of excreta disposal by a household’s members. Next we highlight emerging conditions found for high latrine use, low open defecation activity, and high rates of safe excreta disposal behavior in the study population as measured by SSI and its two components, drawing on significant associations in Table 5.

#### 3.5.1. Conditions for High LUF

LUF scores were positively correlated with increasingly positive attitudes towards using latrines as measured by both the SD and LS attitude scores (ρ = 0.33 and 0.50; *p* = 0.008 and <0.00005, respectively) and with increasing satisfaction with the facility and with its location (linear *p* = 0.013 and 0.005, respectively). Higher LUF was also significantly associated with each tested indicator of greater convenience, proximity, and ease in obtaining water needed for using the latrine and for bathing after defecation. Having a water source inside the latrine or on the property for flushing, anal cleansing, and for bathing appear to be key conditions leading to high household LUF. Where the household bathed at an off-site surface water source (n = 31), mean LUF was significantly lower (52 *vs*. 73; *p* = 0.00005) than when bathing occurred at home or at a nearby public tube well (n = 40). Mean LUF was also significantly higher where men always fetched their own water for defecating in the latrine (n = 56), than where they sometimes, often or always depended on women, children or others in the household to do so for them (n = 15) (68 *vs*. 46; *p* = 0.0005).

**Table 5:** External validity of the Safe San Index and its components: statistical tests of association in the expected direction with conditions for high latrine use and low open defecation behavior (71 households with functioning latrine at F1) (*p*-values < 0.05 in bold).

Condition	Indicator Variable	Description	Mean [SD] or Levels (n)	LUF Association	ODR7 Association	Safe San Index Association
-1-	-2-	-3-	-4-	-5-	-6-	-7-
				**Spearman’s Rho (2-tailed *p*-value)**		
POSITIVE ATTITUDE TOWARD USING LATRINES (2 variables)	1a. Positive attitude SD score	9 semantic differential (SD) items (3 pt scale) summed (range 9–27) (see Suppl. Material)	25.9 [2.2]	0.311	−0.211	0.251
				**(*p* = 0.008)**	(*p* = 0.077)	**(*p* = 0.035)**
	1b. Positive attitude Likert score	44 Likert scale attitudes (3 pt scale) towards using latrines & open defecation (range 0–44) (see Supplementary Material)	37.2 [4.5]	0.495	−0.502	0.542
				**(*p* < 0.00005)**	**(*p* < 0.000005)**	**(*p* < 0.000005)**
				**Mean [SD] (ANOVA and Linear trend *p*-values)**		
SATISFACTION(2 variables)	2a. Satisfaction with toilet	Satisfaction with toilet as place to defecate for household	V. satisfied (6) Satisfied (43) Dissatisfied (19) V. dissatisfied (3)	83.5 [10.9]	0 [0]	83.5 [10.9]
				64.1 [20.5]	27.5 [29.2]	50.9 [28.4]
				59.3 [17.4]	29.1 [26.2]	44.1 [23.4]
				49.0 [32.0]	51.9 [50.1]	31.8 [38.8]
				(*p* = 0.023)	(*p* = 0.056)	(*p* = 0.012)
				**(Lin. *p* = 0.013)**	(Lin. *p* = 0.069)	**(Lin. *p* = 0.012)**
	2c. Satisfaction with location	Satisfaction with toilet location	V. satisfied (7) Satisfied (60) Dissatisfied (4) V. dissatisfied (0)	81.9 [11.0]	0 [0]	81.8 [11.0]
				62.7 [19.7]	28.8 [28.0]	48.3 [27.1]
				45.9 [28.4]	39.6 [48.8]	37.9 [36.2]
				-	-	-
				(*p* = 0.012)	(*p* = 0.028)	(*p* = 0.006)
				**(Lin. *p* = 0.005)**	**(Lin. *p* = 0.035)**	**(Lin. *p* = 0.014)**
CONVENIENCE/ EASE OF WATER NEEDED FOR LATRINE (6 variables)	3a. Toilet has H_2_O connection	Observation of facility	Yes (11) No (60)	81.0 [9.8]	2.3 [7.5]	79.5 [13.0]
				60.4 [20.5]	31.1 [29.5]	45.8 [27.1]
				**(*p* = 0.002)**	**(*p* = 0.002)**	**(*p* < 0.001)**
	3b. Location of water for toilet use	Reported (*i.e.*, flushing, anal cleansing)	Inside toilet (12) Inside compound (38) Outside compound (21)	81.3 [9.8]	2.1 [7.2]	79.9 [12.8]
				62.6 [19.0]	28.1 [24.4]	48.2 [25.0]
				55.2 [25.3]	37.8 [36.7]	39.4 [29.7]
				(*p* = 0.001)	(*p* = 0.002)	(*p* < 0.0005)
				**(Lin**. ***p* = 0.001)**	**(Lin. *p* = 0.001)**	**(Lin. *p* < 0.0001)**
	3c. Location of water for bathing	Reported source location	In dwelling (14) In compound (18) Outside compound (39)	74.6 [10.7]	10.9 [17.1]	66.9 [16.4]
				70.2 [15.5]	21.8 [17.5]	56.9 [22.9]
				56.6 [22.8]	34.5 [34.1]	42.6 [30.9]
				(*p* = 0.004)	(*p* = 0.022)	(*p* = 0.011)
				**(Lin. *p* = 0.001)**	**(Lin. *p* = 0.006)**	**(Lin. *p* = 0.003)**
	3d. Bath at off-site surface source	Combining responses to reported bathing water source and location	Yes (31) No (40)	51.6 [21.0]	38.5 [32.7]	36.3 [28.0]
				72.9 [14.8]	17.4 [22.4]	62.4 [22.8]
				**(*p* < 0.00005)**	**(*p* = 0.002)**	**(*p* < 0.00005)**
	3e. Ease of water access	Perception of ease of fetching water to use toilet	Very easy (34) Not very easy (37)	71.6 [18.2]	17.2 [24.4]	62.3 [26.6]
				56.3 [20.1]	35.3 [30.7]	40.6 [25.8]
				**(*p* = 0.001)**	**(*p* = 0.008)**	**(*p* = 0.001)**
	3f. Men fetch own water for flushing	Reported frequency when males defecate in latrine	Always (56) Not Always (15)	68.3 [18.1]	22.8 [29.1]	56.6 [27.7]
				46.2 [20.5]	40.9 [26.0]	56.6 [27.7]
				**(*p* < 0.0005)**	**(*p* = 0.031)**	**(*p* = 0.001)**
FACILITY FUNCTIONALITY/QUALITY (3 variables)	4a. Fully constructed	Toilet structure considered by user to be fully constructed	Yes (26) No (45)	77.5 [15.3]	12.1 [19.7]	69.7 [21.7]
				55.6 [19.0]	35.0 [30.6]	40.2 [26.0]
				**(*p* < 0.000005)**	**(*p* = 0.001)**	**(*p* < 0.00001)**
	4b. Attached bathroom	Observation of facility	Yes (14) No (57)	75.3 [13.6]	19.4 [35.6]	64.9 [30.6]
				60.7 [21.1]	28.5 [27.4]	47.6 [26.8]
				**(*p* = 0.017)**	(*p* = 0.282)	**(*p* = 0.039)**
	4c.Construction quality	Respondent’s perception of construction quality used to build facility	Excellent (3) Good (27) Fair (34) Poor (5) Very poor (2)	86.1 [14.6]	0 [0]	86.1 [14.6]
				64.7 [22.0]	25.3 [31.7]	53.8 [30.2]
				63.4 [18.4]	26.0 [26.0]	49.5 [25.5]
				61.1 [10.4]	34.4 [25.4]	41.8 [22.3]
				24.1 [18.3]	75.0 [35.4]	9.3 [13.1]
				(*p* = 0.021)	(*p* = 0.072)	(*p* = 0.037)
				**(Lin**. ***p* = 0.011)**	**(Lin**. ***p* = 0.025)**	**(Lin**. ***p* = 0.007)**
OWNERSHIP/VALUATION (6 variables)	5a.Facility investment (latrine type)	Categories according to degree of household self-investment (confirmed)	GOI subsidy (5) GOI subsidy + $ (43) Self-financed (23)	44.4 [16.5]	45.0 [40.2]	28.9 [25.5]
				58.2 [20.0]	33.3 [27.1]	42.5 [25.2]
				77.9 [13.4]	10.0 [23.5]	71.7 [22.5]
				(*p* < 0.00005)	(*p* = 0.002)	(*p* < 0.00005)
				**(Lin. *p* < 0.00001)**	**(Lin. *p* = 0.001)**	**(Lin**. ***p* < 0.00001)**
	5b. Chose design	Household chose design (*vs*. NGO, mason, gov’t)	Yes (37) No (33) * 1 md.	72.0 [15.7]	18.6 [27.8]	61.3 [26.4]
				53.4 [21.1]	36.4 [28.2]	38.3 [24.9]
				**(*p* < 0.0001)**	**(*p* = 0.01)**	**(*p* < 0.0005)**
	5c. Subsidy was reason to build	Reported primary reason for building	Subsidy offer (40) Non-health reason (22) Avoid hookworm (4) * 5 md	55.0 [19.8]	34.2 [27.0]	40.0 [23.8]
				72.8 [16.6]	20.1 [31.0]	61.4 [28.4]
				86.1 [7.4]	0 [0]	86.1 [7.4]
				(*p* < 0.0001)	(*p* = 0.024)	(*p* < 0.0005)
				**(Lin. *p* < 0.00005)**	**(Lin. *p* = 0.007)**	**(Lin. *p* < 0.00005)**
OWNERSHIP/VALUATION (6 variables)	5d. O&M emptying	Latrine has been emptied	Yes (8) No (63)	70.0 [16.7]	8.3 [17.8]	66.6 [21.5]
				62.8 [21.0]	28.9 [29.6]	49.0 [28.5]
				(*p* = 0.355)	(*p* = 0.059)	(*p* = 0.098)
	5e. O&M improvements	Improvements made since built	Yes (10) No (61)	69.1 [12.6]	24.2 [30.5]	54.8 [23.7]
				62.7 [21.6]	27.0 [29.2]	50.4 [29.0]
				(*p* = 0.37)	(*p* = 0.78)	(*p* = 0.75)
				**Spearman’s Rho (2-tailed *p*-value)**		
	5f. Toilet age	Years toilet facility has been in operation and use	7.2 [6.5] * 1 md	0.335	−0.357	0.389
				**(*p* = 0.005)**	**(*p* = 0.002)**	**(*p* = 0.001)**
				**# of Index-predictor variable associations**		
Summary	19 predictor variables of 5 conditions	Index-predictor variable associations in the expected direction:	Not significant	2	5	2
			*p* < 0.05, ≥0.01	3	4	4
			*p* < 0.01, ≥0.001	8	9	5
			*p* < 0.001	6	1	8

Note: * md: missing data.

As facility functionality increased and construction quality improved, LUF scores increased significantly. The presence of an attached bathroom (n = 14) and having a fully constructed structure (n = 26) increased LUF by 23% and 40%, respectively, over no bathroom (n = 57) and not fully constructed (n = 45). LUF scores were significantly associated with four tested indicators of greater sense of ownership and valuation of the facility: having chosen its design, paid fully for its construction, having a main reason for building other than the government subsidy, and years of ownership. Those whose reason for building was to avoid hookworm (n = 4) and those who gave a non-health benefit reason (n = 22) had mean LUF scores of 86 and 73, respectively, compared to 55 for those whose reason was the government subsidy offer (n = 40; *p* < 0.0001).

#### 3.5.2. Correlates of ODR7

Overall, the 19 predictor variables had slightly weaker associations with ODR7 than with LUF, with 3 exceptions: Likert scale (LS) positive attitude scores were more strongly predictive of ODR7 than of LUF, and ODR7 showed a stronger (negative) correlation with past latrine empting and years of ownership than did LUF, possible evidence of habituation to using the latrine with experience and time. Zero ODR7 was reported by 26 households with a functioning latrine (37%), including those who reported being very satisfied with their facility, very satisfied with its location, had as a main reason for building to avoid hook worm, or who perceived its construction quality as excellent (see Table 5). Low mean rates around 10% were observed among households whose source of water and place for bathing after defecation was located at their dwelling (n = 14), who considered their facility fully built (n = 26), whose facility was self-financed (n = 23), or whose latrine had been emptied before (n = 8).

#### 3.5.3. Conditions for High SSI Scores

While SSI scores were associated with all 19 variables tested in the predicted direction, those most strongly associated with SSI were the positive attitude Likert scale score (ρ = 0.54; *p* < 0.000005), both indicators of facility satisfaction, every indicator tested of convenience and ease of obtaining water, two of three tested indicators of facility functionality and quality, and four of six tested indicators of facility ownership and valuation (see Table 5). SSI which integrates ODR7 and LUF was more strongly correlated positively with years of latrine ownership, than was LUF alone.

## 4. Discussion and Conclusions

There are few low-cost and reliable approaches to accurately measure consistent latrine use or open defecation behavior. Here we present the Safe San Index (SSI), a self-report survey-based instrument developed to measure and quantify the degree of safety of excreta disposal behavior of households in the Indian context. Standardized on a scale from 0 (0% safe) to 100 (100% safe), SSI is a composite index of recent open defecation activity (the ODR7 score) and safe latrine use behavior (the LUF score) which accounts for individual behavior and a full range of domestic human excreta disposal and defecation occasions important for health. The SSI and its two components offer practitioners, researchers and policy-makers a low-cost and reliable approach to quantify the consistency of latrine usage and measure progress towards safe excreta disposal in India.

We observed high correlation and agreement of repeat measurements of household ODR7, LUF, and SSI under stable household behavioral conditions, and significant differences in household scores where household behavioral conditions changed, providing evidence in both directions of robust reliability of the SSI and its two component scales. No correlation was observed between a household’s ODR7, LUF, and SSI scores and the respondent’s Social Desirability score, demonstrating the ability of the SSI and its components to provide socially unbiased quantitative estimates of open defecation activity and latrine use behavior in a typical rural Indian population. Lack of social bias in index scores can be attributed in part to the use of multiple items and closed-ended formulations. Questions were specific to individual household members and specific situations, and responses restricted to choosing 1 of 4 ordered options. Together these elements help limit imprecise responses and reduce bias [35]. Social bias is also reduced by the multi-item computational approach, where weight given to the respondent’s self-rated behavior is balanced by her rating of like-members’ and others’ behaviors in computing the LUF and ODR7 scores. We found respondents somewhat more likely to report “never” open defecating during the last seven days than they reported “never” for others of their age and marital status in their household, with this tendency observed mainly among female respondents (see Figure 3b). Low observed social bias, especially in relation to ODR7, may be unique to India where open defecation is widely practiced with little stigma [4]. The relative importance of these explanations is unknown. If social stigma around open defecation were to increase substantially in India in the future, the ability of the SSI to produce socially unbiased scores would need to be re-evaluated.

A household’s propensity to use the latrine (their LUF score) and the reported amount of open defecation activity of its members in the preceding seven days (their ODR7 score) did not necessarily strongly correlate, providing new insight into large differences in open defecation activity among households with similar reported tendencies of using their latrine, and evidence of temporal and individual variability in household defecation behavior in the study population. For example, among latrine owners with LUF scores in the “sometimes use” range, ODR7 varied from 0 (no open defecation) to 100 (100% of members defecating openly at least once on each of the preceding seven days), resulting in a 60 point range in SSI scores among “sometimes” use latrine owning households.

In just 3% (95% CI 0.5% to 8%) of study households with a functioning improved latrine was the facility consistently used by all members at all defecation and feces disposal occasions (*i.e.*, SSI = 100). This is considerably lower than reported in rural Puri District based on less precise methods of assessing latrine use [10]. Overall, the mean SSI score among latrine owners was 51 (95% CI 44 to 57), meaning only about half of excreta disposal occasions were safely disposed, with rates significantly lower among those with a government subsidized latrine (SSI = 41; 95% CI 39 to 48) compared to those with a self-financed latrine (SSI = 72; 95% CI 63 to 81). On any given day at the time of the survey, an estimated 27% (95% CI 20% to 33%) of the study population with access to a functioning household latrine none-the-less defecated in the open. There was some evidence to suggest this proportion may reach higher levels in the post-harvest season.

Trends were found of which demographic categories were more likely to use the latrine as average household propensity to use (LUF score) increased. Half of our sample of latrine owners fell into the “mostly” use LUF score range and reported 100% consistent latrine use among >70% of adult members, the elderly, and school age children in their household, at each occasion of the day and night. The other half showed much lower rates of consistent use by members, with the exception of school age children, the only demographic to remain above 50% even among “rarely/never” use latrine owning households. Our results confirm what other studies in India have often observed, that men’s and elders’ rates of consistent latrine use tend to be lower than women’s [4,6,10,25]. Further, we found some evidence of a disparity in women’s use depending on their marital status, with married compared to unmarried women more likely to use latrines, and women’s usage varying with the time of the day, with use at mid-day and evening lower, compared to morning, among latrine owners who on average “sometimes” used their latrine in this study.

In nearly all latrine owner households, there was very low consistent use for the safe disposal of feces of young children and of ill persons, populations whose behaviors typically receive little direct attention in sanitation programs, but which may account for a disproportionate share of infectious pathogens in the community. These findings should indicate where behavior change and educational efforts need to be focused as part of latrine promotion programs.

All five *a priori* conditions for high latrine use (Figure 1) identified from literature were observed as important determinants of rates of safe excreta disposal behavior in the study population. Household LUF and SSI scores were positively correlated with increasingly positive attitude towards latrine use and with increasing satisfaction with the facility and with its location. To our knowledge, this is the first time attitudes towards using latrines have been quantified and shown to measurably affect open defecation and latrine use behavior in India, pointing to the potential value of investing in strategically designed behavior change communications. Very convenient and easy on-site access and proximity to water for the latrine and for bathing at home after defecation appear to be critical for high LUF, low ODR7, and high SSI. Water access was also associated with latrine usage in rural Uttarakhand, India [5]. Functionality, construction quality, and sense of ownership and valuation of the latrine facility by the household were each significantly associated with higher household LUF and lower ODR7 scores. Studies in other low-resource settings have reported sub-optimal latrine use associated with poor latrine quality, lower functionality, lack of self-investment, unclean latrines, and other limitations on access [20,21,25,28,32,33,34]. On the other hand, continued open defecation in India and elsewhere has widely been attributed to “mind-sets”, cultural preferences, and social norms [4,6,9,10]. Thus, a further value to measuring both LUF and ODR7 as distinct dimensions of safe excreta disposal is that their key determinants may be somewhat different.

While other Indian studies, including evaluations of the government’s TSC program, consistently find inconsistent latrine use both within and across latrine owning households [5,6,7,10], measurement methods to date have precluded estimating the amount of continued open defecation at the individual level or pinpointing which demographic groups and practices are likely to contribute most to continued fecal contamination of a community. If safe excreta disposal is the ultimate goal of sanitation investments, then a reliable, rapid, and accurate means of assessing and measuring it is necessary for evaluating and improving the design of a wide range of sanitation investment strategies. 

The SSI and its ODR7 and LUF components provide new metrics to measure outcomes from Community Led Total Sanitation and other behavior change approaches that seek to end open defecation and unsafe excreta disposal behavior in rural India, such as the Indian government’s Nirmal Gram Puraskar and Nirmal Bharat Abhiyan strategies. The metrics and methodology may have value elsewhere where sanitation programs would benefit from standardized and reliable measurement of changes in household open defecation activity and safe disposal behavior. Given the small size of this pilot study, and potential for conditions to vary across rural India, it would be worthwhile to test the SSI’s application more widely with a larger sample of rural households to further establish its utility and draw definitive conclusions about specific factors associated with sanitation behavior observed in this study’s small sample. In new applications, pretesting is recommended as some adjustment to item content may be needed.

Non-standard latrine use measurement methods currently employed in the sector contribute to variability in reported usage rates, and prevent tracking progress over time or comparing between program approaches. The SSI improves on sector practice of using self-report questions to assess household latrine use and open defecation behavior in several ways. First it measures behavior at the individual level and quantifies its consistency and safety. Also unique is the integration of open defecation, latrine use, and disposal behaviors into a coherent composite index with a scale spanning from completely unsafe to completely safe behavior. With the advantage of demonstrated reliability and validity, the SSI offers a standard method of quantifying safe excreta disposal within and between contexts and communities, as well as tracking over time. The approach requires a larger number of questions than is currently used in typical household surveys of sanitation access and use, but the questions, responses and computational effort to compute each component and the overall SSI score are straightforward.

There are several limitations to the SSI. The approach depends on the household respondent knowing with reasonable accuracy the daily defecation routines and habits of members in their household residing at home. In much of rural India, household latrines are typically located in a private area behind the house where household chores are done. Women, particularly married women, spend much of their day confined to the home and are responsible for latrine cleaning and upkeep along with other cooking, childcare, and cleaning responsibilities. In this context, it is plausible that an Indian married woman would have good knowledge of cohabitating family members’ and children’s defecation routines, including use of the household latrine/toilet. However, inability to test accuracy of respondents’ knowledge of their family members’ home-based defecation behavior is a limitation of the measurement approach, albeit one that is common to many household-based self-report surveys of sanitation access and usage. Because we tested for and found no evidence of biased reporting of defecation behavior, any SSI error arising from respondents’ imperfect knowledge is likely to be unbiased. Defecation behavior when traveling away from home or when children are at school is beyond the scope of the SSI which focusses on defecation behavior within the control of the household. Finally, the SSI has been designed specifically for application in India. As such, the demographic categories for assessing use, derived empirically, reflect the socio-cultural context of Indian society. Before application and validation for use beyond India, socio-culturally appropriate demographic categories for SSI items would need to be developed.

Global and other official estimates of open defecation rates for India may be considerably underestimated as these are based largely on latrine coverage and imprecise questions about use. This pilot study applying the SSI shows high rates of continued open defecation persist among many categories of rural latrine owners in India, but are particularly likely for the 95 million government-subsidized rural household latrines built under the TSC. Open defecation rates may be particularly high where water for anal cleansing, flushing and post-defecation bathing is not readily available inside the latrine or on the property, attitudes towards open defecation have not changed, facility functionality and construction quality are perceived to be inadequate for habitual consistent use, a strong sense of facility ownership and value is lacking, and facility satisfaction is low. Despite a shift from emphasis on latrine construction in prior government programs to ending open defecation in the TSC, more accurate measurement of excreta disposal behavior using the SSI in this study suggests India may have a considerable way still to go to achieve consistent use and safe excreta disposal among many latrine owners.

## Supplementary Files

Supplementary File 1
