# Supplementary material for: Measuring the Safety of Excreta Disposal Behavior in India with the New Safe San Index: Reliability, Validity and Utility
Source: Int J Environ Res Public Health. 2014 Aug 15;11(8):8319–46. doi: 10.3390/ijerph110808319 (PMC4143864; doi:10.3390/ijerph110808319)
Supplement: Supplementary File 1 [file ijerph-11-08319-s001.pdf]

Measuring the Safety of Excreta Disposal Behavior in India with the New Safe San Index: Reliability, Validity and Utility

---

Latrine Usage Attitude Measurement

Items were based directly on content from preliminary focus group discussions on latrines, their usage and open defecation behavior with latrine owners and with NGO field staff working on latrine promotion and construction in rural communities, as part of the Indian Government Total Sanitation Campaign in Odisha India, and finalized after pretesting.

Table S1 lists the 9 semantic differential attitude measurement items, response scale, and scoring. Verbal instructions for the semantic differential measurement task which were read to the respondent were as follows: "We would like to know your personal feeling about using a toilet for daily defecation. After we read each pair of words, please pick the word that is closest to how you (would) feel about using a toilet for daily defecation compared to going for open defecation. If you are unsure how you feel, you can say 'Not sure' or 'Don't know'." Individual item response scores were summed to produce a total attitude score, with a possible range from 9 (all negative) to 27 (all positive).

Table S2 lists the 44 Likert scale agree-disagree items, response scale, and scoring. An original list of 50 agree-disagree statements was sent to a panel of 3 Indian and 4 International experts on rural sanitation promotion and latrine usage behavior in India. Each expert was asked to evaluate each statement and decide if it reflected a positive attitude or likelihood of using a latrine, a negative attitude/likelihood of using a latrine, or was ambiguous. Statements rated as ambiguous by 2 or more experts, or those for which more than 1 expert disagreed were removed, resulting in the final 44 statements. Verbal instructions for the Likert attitude measurement task were as follows: "We are going to say some statements and we want you to know if you agree or disagree. For example, if I said, 'rice is grown in the field, not on a tree' you would say 'I Agree'. If instead I said, 'a motorcycle has 4 wheels' you would say 'I disagree'. Do you have any questions?" As in the semantic differential attitude scoring, individual item responses were summed to produce a total attitude score, with a possible range from a low of 0 (all negative) to a high of 44 (all positive).

Table S1. Semantic difference items measuring positive *vs*. negative attitudes towards using a latrine for defecation to validate the Safe San Index in the Odisha, India pilot study.

#	Statement preamble	Semantic differential pair (value)	Mean LUF and ODR7 behavior for those with Pos.(3)/Neg.(1) attitude item response(ANOVA lin. *p* value)	
			LUF	ODR7
1	"Using a household toilet for daily defecation is…	Holy (3)…Unholy (1)…Not sure (2)	64.1/37 (0.06)	25.7/50 (0.17)
2	Ditto	Smelly (1)…Not smelly (3)…Not sure (2)	64.1/51.5 (0.12)	25.2/42.7 (0.11)
3	Ditto	Private (3)…Exposed (1)…Not sure (2)	64.1/24.1 **(0.007)**	25.6/75 **(0.017)**
4	Ditto	Complicated (1)…Simple (3)…Not sure (2)		
5	Ditto	More acceptable (3)…Less acceptable (1)…Not Sure (2)	66.3/33.1 **(0.001)**	23.8/55.6 (0.024)
6	Ditto	Decreases contamination (3)…Increases contamination (1)…Not sure (2)	64.2/37.0 **(0.040)**	25.0/50 **(0.033)**
7	Ditto	Enjoyable (3)…Not enjoyable (1)...Not sure (2)	63.7/37.0 (0.10)	26.2/50 (0.24)
8	Ditto	More healthy (3)…Less healthy (1)…Not sure (2)	69.0/43.4 (0.06)	25.9/45.4 (0.18)
9	Ditto	Protects environment (3)…Destroys environment (1)…Not sure (2)	63.3/50.2 (0.31)	26.4/44.4 (0.30)
**Total score: (sum item values)**		**Possible range: 9–27 points; Mean = 25.9, St. Dev = 2.20 (n = 71 w/latrine)**		

Table S2. Likert scale agree-disagree attitude statements measuring positive *vs*. negative attitudes towards using a latrine for defecation in the Odisha, India Safe San Index pilot study.

#	Likert scale attitude statement	Rating (Value)
1	Having a latrine for the newly married wife does not disturb the routines of the other household members.	Agree (1) Disagree (0) Not sure (0.5)
2	Storing feces in the form of a pit on the house property is unholy	Agree (0) Disagree (1) Not sure (0.5)
3	I enjoy the freedom ("khola") of going for open defection	Ditto
4	It is inconvenient to get the water needed to defecate in the toilet at night.	Ditto
5	I have been using a latrine for defecation since my childhood	Agree (1) Disagree (0) Not sure (0.5)
6	Using a latrine to defecate protects the environment from contamination.	Agree (1) Disagree (0) Not sure (0.5)
7	Latrine protects me from getting caught in the rain.	Agree (1) Disagree (0) Not sure (0.5)
8	The location of the latrine is convenient for use at night.	Agree (1) Disagree (0) Not sure (0.5)
9	The family's prestige is hampered if the women go for open defecation.	Agree (1) Disagree (0) Not sure (0.5)

Table S2. *Cont.*

#	Likert scale attitude statement	Rating (Value)
10	I use the latrine because I am afraid of snakes, leeches and other kinds of insects.	Agree (1) Disagree (0) Not sure (0.5)
11	Defecating in the open is good for the environment.	Agree (0) Disagree (1) Not sure (0.5)
12	Regular use of the latrine for defecation would restrict me to the house all day.	Ditto
13	It is unsafe for children to use the latrine.	Ditto
14	Taking a stroll in the evening is an important benefit of going for open air defecation.	Ditto
15	In our community, it is easy to find a private place where I can go for open defecation.	Ditto
16	There are too many people in the household for just one latrine.	Ditto
17	Financing for upkeep of the latrine is part of our household budget.	Agree (1) Disagree (0) Not sure (0.5)
19	An important reason for us to have a latrine is for when guests visit.	Ditto
20	My morning routine is not suited for (fit well with) for using the latrine to defecate.	Agree (0) Disagree (1) Not sure (0.5)
21	Most of the men in my village are regular users of the latrine throughout the year.	Agree (1) Disagree (0) Not sure (0.5)
22	I enjoy going for open defection in the evening to relax and distress from household chores.	Agree (0) Disagree (1) Not sure (0.5)
23	It is more work for the mother when the child defecates in the latrine.	Ditto
24	The latrine's location provides privacy for defecation during the day.	Agree (1) Disagree (0) Not sure (0.5)
25	For the work I do, it is more suitable to go outside the house to defecate.	Agree (0) Disagree (1) Not sure (0.5)
26	The daughter in law and father in law should not use the same latrine.	Ditto
27	After our latrine was built, the whole family started using it right away.	Agree (1) Disagree (0) Not sure (0.5)
28	The pit is small and would fill too quickly if everyone used the latrine every day.	Agree (0) Disagree (1) Not sure (0.5)
29	In the morning if the latrine is in use by another member, I go for open defecation.	Ditto
30	We sometimes use the latrine for storing dung or wood or other fuel.	Ditto
31	The NGO was responsible for building the latrine, so they should improve it.	Ditto
32	Most of my friends and relatives use the latrine every time they defecate.	Agree (1) Disagree (0) Not sure (0.5)
33	Getting hookworm is a concern for our family when going to open defecation.	Ditto
34	When land and water is freely available, I don't find it necessary to spend money for a toilet.	Agree (0) Disagree (1) Not sure (0.5)
35	To dispose of small child's feces in the latrine is too complicated.	Ditto
37	I dislike the feeling of confinement ("bandhi heba") when I defecate in a latrine.	Ditto
38	Open defecation is part of my every day activities.	Ditto

Table S2. *Cont.*

#	Likert scale attitude statement	Rating (Value)
39	We chose the design for this latrine after considering different styles.	Agree (1) Disagree (0) Not sure (0.5)
40	Accessing water for bathing after latrine use is difficult.	Agree (0) Disagree (1) Not sure (0.5)
41	If my latrine stopped functioning I would construct a new one.	Agree (1) Disagree (0) Not sure (0.5)
42	In this community we fear for the security of women when they go for open air defecation.	Agree (1) Disagree (0) Not sure (0.5)
43	Children should always defecate in the latrine.	Ditto
44	Using the latrine for defecation saves time.	Ditto
45	I worry about bringing germs from the open defecation are back to the home on my feet.	Ditto
46	It is more convenient (“sahaja”) in the morning to use the latrine because of my busy schedule than going for open air defecation.	Ditto
47	We feel embarrassed when outsiders come to our village and see the feces on the roadside. (unanimous 120 all agreed)	Not included in total
**Total score: sum item values. Possible range: 0 to 44 points.**		

© 2014 by the authors; licensee MDPI, Basel, Switzerland. This article is an open access article distributed under the terms and conditions of the Creative Commons Attribution license (http://creativecommons.org/licenses/by/3.0/).
